# Development of an Automated Free Flap Monitoring System Based on Artificial Intelligence

**DOI:** 10.1001/jamanetworkopen.2024.24299

**Published:** 2024-07-26

**Authors:** Jisu Kim, Sang Mee Lee, Da Eun Kim, Sungjin Kim, Myung Jin Chung, Zero Kim, Taeyoung Kim, Kyeong-Tae Lee

**Affiliations:** 1 Department of Plastic Surgery, Samsung Medical Center, Sungkyunkwan University School of Medicine, Seoul, South Korea; 2 Department of Health Sciences and Technology, Samsung Advanced Institute for Health Sciences & Technology, Sungkyunkwan University, Seoul, South Korea; 3 Medical AI Research Center, Research Institute for Future Medicine, Samsung Medical Center, Seoul, South Korea; 4 Banobagi Plastic Surgery Clinic, Seoul, South Korea.; 5 Department of Data Convergence and Future Medicine, Sungkyunkwan University School of Medicine, Seoul, South Korea; 6 Department of Radiology and Medical AI Research Center, Samsung Medical Center, Seoul, South Korea

## Abstract

**Importance:**

Meticulous postoperative flap monitoring is essential for preventing flap failure and achieving optimal results in free flap operations, for which physical examination has remained the criterion standard. Despite the high reliability of physical examination, the requirement of excessive use of clinician time has been considered a main drawback.

**Objective:**

To develop an automated free flap monitoring system using artificial intelligence (AI), minimizing human involvement while maintaining efficiency.

**Design, Setting, and Participants:**

In this prognostic study, the designed system involves a smartphone camera installed in a location with optimal flap visibility to capture photographs at regular intervals. The automated program identifies the flap area, checks for notable abnormalities in its appearance, and notifies medical staff if abnormalities are detected. Implementation requires 2 AI-based models: a segmentation model for automatic flap recognition in photographs and a grading model for evaluating the perfusion status of the identified flap. To develop this system, flap photographs captured for monitoring were collected from patients who underwent free flap–based reconstruction from March 1, 2020, to August 31, 2023. After the 2 models were developed, they were integrated to construct the system, which was applied in a clinical setting in November 2023.

**Exposure:**

Conducting the developed automated AI-based flap monitoring system.

**Main Outcomes and Measures:**

Accuracy of the developed models and feasibility of clinical application of the system.

**Results:**

Photographs were obtained from 305 patients (median age, 62 years [range, 8-86 years]; 178 [58.4%] were male). Based on 2068 photographs, the FS-net program (a customized model) was developed for flap segmentation, demonstrating a mean (SD) Dice similarity coefficient of 0.970 (0.001) with 5-fold cross-validation. For the flap grading system, 11 112 photographs from the 305 patients were used, encompassing 10 115 photographs with normal features and 997 with abnormal features. Tested on 5506 photographs, the DenseNet121 model demonstrated the highest performance with an area under the receiver operating characteristic curve of 0.960 (95% CI, 0.951-0.969). The sensitivity for detecting venous insufficiency was 97.5% and for arterial insufficiency was 92.8%. When applied to 10 patients, the system successfully conducted 143 automated monitoring sessions without significant issues.

**Conclusions and Relevance:**

The findings of this study suggest that a novel automated system may enable efficient flap monitoring with minimal use of clinician time. It may be anticipated to serve as an effective surveillance tool for postoperative free flap monitoring. Further studies are required to verify its reliability.

## Key Points

**Question:**

Is it feasible to develop an automated free flap monitoring system using artificial intelligence–based deep learning techniques, requiring minimal human intervention?

**Findings:**

In this prognostic study, models capable of automatically segmenting the flap area from images and detecting anomalies in flap perfusion status based on the captured images were developed. The integrated automated flap monitoring system demonstrated effective application in a clinical setting.

**Meaning:**

This study suggests that an artificial intelligence–based automated system may enable efficient flap monitoring with minimal use of clinician time.

## Teaser

This prognostic study investigates an automated free flap monitoring system that uses artificial intelligence (AI)–based deep learning techniques, minimizing human involvement for assessment of flap perfusion status.

## Introduction

Free tissue transfer using microsurgery has emerged as a popular technique for managing diverse types of defect reconstruction. Despite a recent steep increase in its success rate, postoperative flap compromise still occurs in 3% to 10% of cases,^1^ possibly leading to flap failure. Early detection of compromised flaps with timely intervention has been shown to increase the likelihood of successful salvage.^2^ Therefore, close postoperative monitoring is critical to achieve optimal results.

The most common method for monitoring free flaps is physical examination, such as inspecting flap color and checking temperature.^3,4^ Despite the development of alternative monitoring methods, their routine clinical use remains limited. Consequently, clinical assessment by trained personnel remains the criterion standard due to its reliability. However, it requires significant clinician time for frequent monitoring, as it is typically conducted every 2 to 3 hours or even hourly during the early postoperative period. This approach places a considerable administrative burden on medical caregivers and consumes significant resources.^5,6^ With the improvement in free flap survival rates, meticulous monitoring is often limited to confirming flap viability, with compromised flap cases rarely encountered, potentially making the process less cost-effective.^7^

Currently, the use of artificial intelligence (AI) has been increasingly gaining attention across various fields, including plastic surgery. However, its application to flap monitoring remains limited. A few studies have applied AI to assess flap status based on photographs, capillary refilling time, and temperature. Although these studies show promising reliability,^8,9,10^ they may not fully address the issue of significant use of medical resources, as the flaps still require physical assessment by medical personnel.

A fully automated flap monitoring system requiring very limited human involvement as well as having accuracy and reliability in evaluating flap perfusion status as high as that with clinical monitoring could be an ideal tool for free flap monitoring. Although achieving a higher accuracy than clinical examinations with an automated flap monitoring system may be challenging, its development, functioning as a sentinel by autonomously conducting surveillance and promptly alerting medical staff if any abnormal signs are detected, could lead to a transformative change in flap monitoring. The purpose of this study was to develop a new flap monitoring system, which is designed to automatically perform flap monitoring with the aid of AI, minimizing the need for human effort while still being able to evaluate flap perfusion status reliably and accurately.

## Methods

### Scheme of This Project

**Figure 1.**
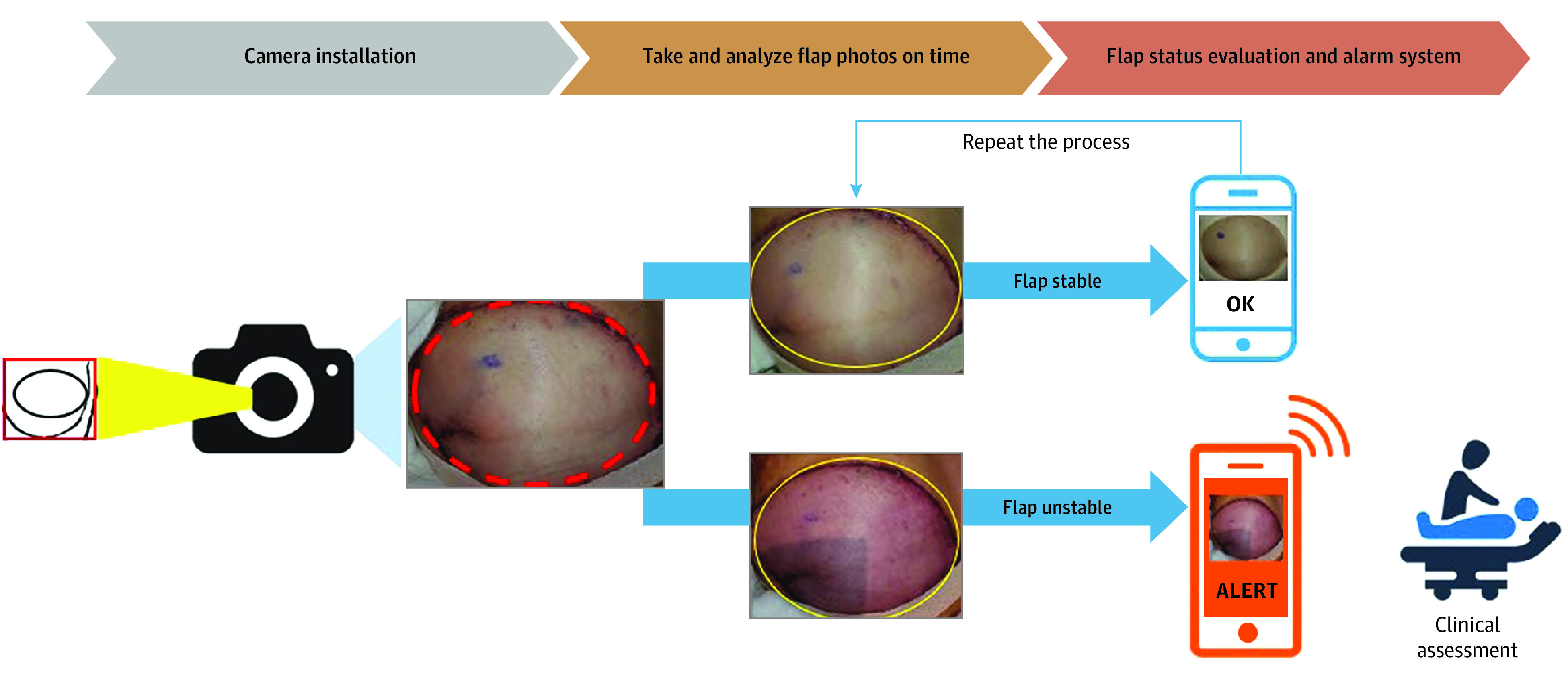
Scheme of the Artificial Intelligence–Based Automated Flap Monitoring System First, a smartphone camera with a monitoring application was installed in a place where the flap could be well observed, and the camera automatically took photographs at regular intervals. The system automatically identified the flap area from the images and evaluated its perfusion status. If there were no issues, the process would be repeated. If an abnormality was detected, the medical staff assigned in advance was called to evaluate the flap status directly.

The system we aimed to develop (Figure 1) required the creation of 2 essential programs. First, a segmentation model was required to identify the flap area accurately and automatically in captured photographs. This capability was crucial for system automation, particularly in clinical scenarios in which the flap positioning might vary due to patient movement. Second, another program was needed to assess the perfusion status of flaps based on the segmented image and to detect any abnormal signs that may arise. This study was conducted after obtaining approval from the institutional review board of the Samsung Medical Center. Patients provided oral consent to participate. This study followed the Standards for Reporting of Diagnostic Accuracy (STARD) reporting guideline.

### Study Materials

Photographs from the conventional flap monitoring were used, categorized into retrospective and prospective subsets. Initially, retrospective photographs were gathered from patients who underwent free flap–based reconstruction between March 1, 2020, and February 28, 2022. These photographs, taken by several different ancillary physicians using diverse smartphone models, focused primarily on the skin paddle of the flap. Following the monitoring protocol, approximately 15 to 20 photographs were taken per patient. A standardized image capture protocol was lacking, leading to variations in image size, distance, and lighting conditions.

To address these issues, prospective data collection followed a standardized protocol. From March 1, 2022, to August 31, 2023, additional data were collected from patients undergoing free flap reconstruction. A uniform smartphone was used for photo capture under controlled lighting conditions. Images were taken from 5 angles during each session (frontal, superior, inferior, left lateral, and right lateral) at a consistent distance to ensure accurate identification of the flap margin and surrounding area. In addition, wide shots were captured to facilitate recognition of surrounding anatomy and accurate flap detection in various clinical scenarios (eFigure 1 in Supplement 1).

### Data Processing

For the development of a flap segmentation model, hand annotation was performed along the flap margin in the recruited photographs depicting the appearance of the flap. Images with a well-distinguished flap margin were chosen, as they were deemed suitable for constructing and training the model. To develop the second model for grading flap perfusion status, the collected photographs were labeled based on clinical evaluation by 2 independent surgeons (J.S.K. and K.-T.L.) into 2 categories: normal and abnormal. Both surgeons, who specialized in microsurgical reconstruction and had several years of clinical experience, independently evaluated all photographs during the labeling process. Discrepancies, although rare, were resolved through discussions, leading to a consensus in all cases. Cases presenting challenges in assessing flap perfusion status from photographs, including those with discoloration due to slight hematoma, bruising, or partial discoloration extending mostly beyond reliable perfusion territory, were excluded from model training. In addition, images from cases with multiple flaps were excluded due to potential limitations in accurately segmenting all the flaps with the segmentation model.

### Development of Models

#### Segmentation Model

To develop the flap segmentation models showing the best performance, 3 different convolutional neural network (CNN)–based architectures were tested. Two of these architectures were constructed as the backbones for UNet^11^ and VGG16-UNet,^12^ and the third one was a custom CNN architecture, FS-Net (Flap Segmentation Network), with an encoder-decoder structure. The encoder compressed input images (256 × 256 pixels) into a compact 16 × 16-pixel representation through successive 2-dimensional (2D) convolutional layers with batch normalization and ReLU (rectified linear unit) activation functions. After that, the decoder mirrored the encoder’s structure but used 2D transposed convolutional layers to perform upsampling, restoring the spatial resolution back to the original size (eFigure 2 in Supplement 1). The input consisted of 2D images resized to a uniform size of 256 × 256 pixels, and the pixel values of each image were scaled using minimum-maximum normalization. We conducted a 5-fold cross-validation using a random split for the entire dataset that was manually annotated by experts. The parameter settings for each model are as follows: training was conducted with a batch size of 8, a learning rate of 0.001, 200 epochs, a cross-entropy loss function of binary cross entropy, and Adam optimization.

#### Classification Model

To assess the optimal flap status grading model, we evaluated 4 commonly used deep learning models for classification: VGG16,^13^ ResNet50,^14^ InceptionV3,^15^ and DenseNet121.^16^ In addition, a customized CNN model was constructed and compared. The model was structured with 3 convolutional blocks; each block included a 2D convolutional layer with ReLU activation and a maximum pooling layer. After passing through these blocks, the network output was flattened and processed through a dense layer with dropout. Finally, a softmax layer was activated for predicting flap abnormalities. Adam optimization was used as the optimizer and binary cross-entropy was used as the loss function. We initialized the weights for these models and trained them from scratch, without using the frozen values from models pretrained on ImageNet. The input data for the grading models consisted of the results from the flap segmentation models, which were zero-masked except for pixels corresponding to the flap. We split the dataset into a training set and test set under the condition that each patient’s data were included in either the training set or test set. The photographs used for the development of the segmentation model were also included in the training set for the classification model. To validate the model from segmentation to grading, the test set for grading models was completely independent from training for the segmentation model. We used data augmentation for the training set to handle heterogeneous input data with varying view angles, lighting intensities, and contrast levels. The methods included horizontal and vertical flips, along with random adjustments to image brightness and contrast. Detailed parameters for these augmentation techniques are provided in eTable 1 in Supplement 1. The parameter settings for training were as follows: batch size was 16, learning rate was 0.0001, epoch was 13, and optimizer was Adam. We applied the early stopping during training to find the optimal performance, with a patience value of 10 epochs. Afterward, we used Grad-CAM (Gradient-Weighted Class Activation Mapping) to visualize and highlight the regions where the model classified abnormalities in the photographs of the flap.

### Development of Application and Feasibility Assessment of Clinical Application

After completing the development of the 2 models, an application was created to operate on smartphones. The application was designed with a pipeline for automatically capturing flap photographs, performing flap segmentation on the images, grading the perfusion status, and repeating the process if the result was normal. If an abnormal result was detected, the application sent a notification to medical staff. The photo capture interval could be adjusted depending on the situation. Automatic flash activation ensured that flap photographs were captured effectively even in low-light environments, enabling successful segmentation. In addition, we integrated a feature that triggered an alarm message instructing the patient to reposition the surgical site in front of the camera and recaptured images automatically if the flap was not well captured or if the margin was less distinguished, potentially leading to ineffective segmentation. To assess its applicability and performance in real clinical settings, we conducted flap monitoring using the application for patients who underwent free flap–based reconstruction in November 2023 and provided consent for the application.

### Statistical Analysis

The statistical analysis and machine learning process were conducted under a Python, version 3.7, environment, including Pandas, Tensorflow, and Scikit-Lean (Python Software Foundation). Several performance metrics were evaluated using the following measurements: Jaccard index, Dice similarity coefficient, accuracy, area under the curve, sensitivity, and specificity.

## Results

We obtained a comprehensive dataset of 12 395 photographs from a total of 305 patients (median age, 62 years [range, 8-86 years]; 178 male patients [58.4%] and 127 female patients [41.6%]; 302 Asian patients [99.0%] and 3 White patients [1.0%]). Diverse kinds of flaps were used, of which the anterolateral thigh flap (144 patients [47.2%]) was the most commonly used (eTable 2 in Supplement 1).

### Flap Segmentation Using Deep Learning Models (Segmentation Model)

**Figure 2.**
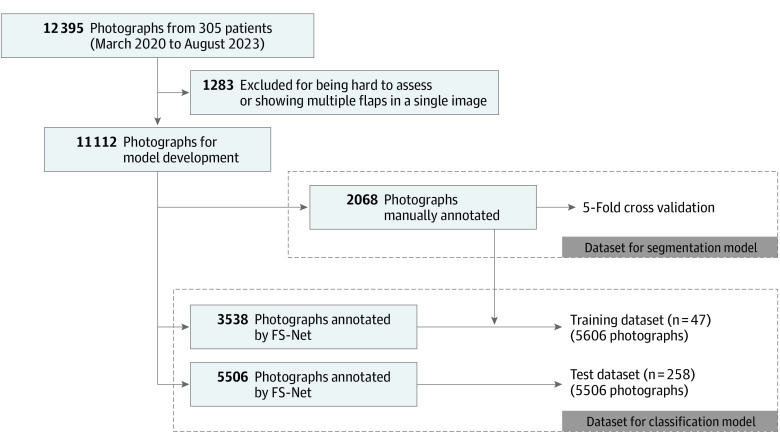
Flowchart of Data Processing FS-Net indicates Flap Segmentation Network.

Figure 2 depicts how the data were used in the development of each model. A total of 2068 photographs showing flap margins clearly were used. Although all models achieved a Dice similarity coefficient of more than 0.95, the FS-Net model demonstrated the most promising results, achieving a mean (SD) Dice similarity coefficient of 0.970 (0.001) (eTable 3 in Supplement 1). The segmentation inference time with FS-Net was less than 0.01 seconds per image on a graphics processing unit machine. eFigure 3 in Supplement 1 depicts a representative example of the result of the FS-Net model processing.

### Flap Status Evaluation Model Using AI-Based Image Classification (Classification Model)

After excluding 1283 of 12 395 photographs based on the above criteria (see Data Processing subsection), 11 112 were used, most of which represented normal flaps (10 115 [91.0%]). The remaining 997 photographs (9.0%) represented abnormal perfusion status of flaps.

**Figure 3.**
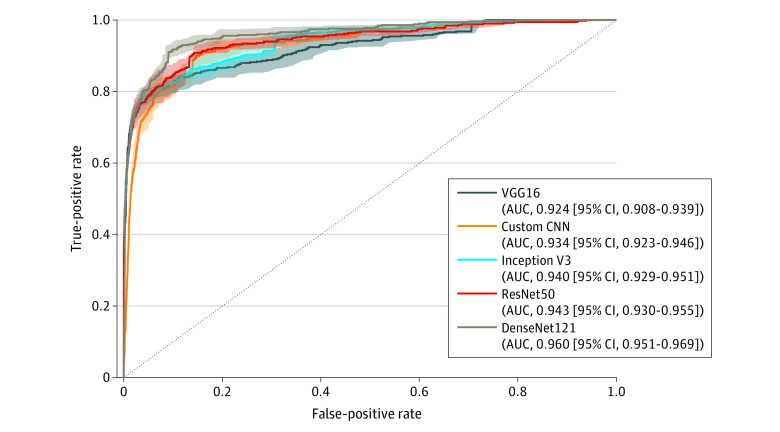
Receiver Operating Characteristic Curve of 5 Grading Models AUC indicates area under the curve; CNN, convolutional neural network.

Of the 11 112 photographs, 5606 were used for constructing and training the models and the other 5506 were used for testing them. Figure 3 shows the performance of the 5 models based on the area under the receiver operating characteristic curve, with DenseNet121 achieving the highest score of 0.960 (95% CI, 0.951-0.969), calculated from 1000 bootstrapped samples. Comparing all models with a similar level of sensitivities of more than 0.9, DenseNet121 showed the highest specificity (eTable 4 in Supplement 1). We conducted an additional analysis to assess the performance of DenseNet121 according to defect site and the type of flap used, and found relatively high performance across all subgroups (eTable 5 in Supplement 1).

The Table presents the confusion matrix for DenseNet121 on the test set, with the threshold set to a probability of 17.8% based on probabilities calibrated through isotonic regression. We observed 457 images of false positives and 45 images of false negatives. Most false positive images (416) arose from cases in which clinical evaluation for flap perfusion might have been warranted due to discoloration related to bruising despite the perfusion being adequate. The remaining 41 instances were attributed to segmentation errors. Analyzing the 45 false negatives, more than half (28 cases) were associated with segmentation errors, particularly in the cases of relatively small flaps used for finger or toe defect coverage, in which neighboring digits were inadvertently included in the segmentation. Among the remaining 17 cases, 7 failed to detect the early phase of arterial insufficiency, while 10 failed to detect the early phase of venous insufficiency.

**Table.** Confusion Matrix for DenseNet121 on the Test Set of 5506 Photographs

Actual value	Predicted value	
	Abnormal	Normal
Abnormal	455 (True positives)	45 (False negatives)
Normal	457 (False positives)	4549 (True negatives)

**Figure 4.**
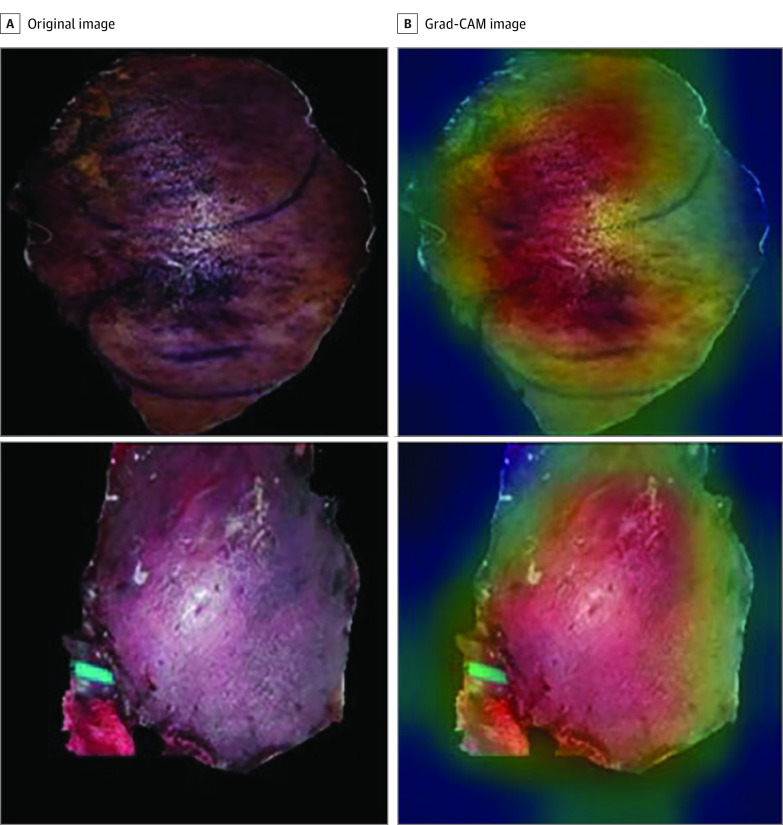
True-Positive Images From the DenseNet121, Visualized Using Gradient-Weighted Class Activation Mapping (Grad-CAM) to Highlight Areas Where the Model Affected Its Decision The images on the left show the appearance of abnormal flap perfusion, which was also identified as abnormal by the classification model (true positive). In the Grad-CAM images on the right, the red areas indicate the regions that the classification model primarily relied on to make its decision.

Of the images used for testing, there were a total of 500 abnormal images, with 98 cases attributed to arterial insufficiency and 402 to venous insufficiency. For arterial insufficiency, 91 of 98 cases were correctly identified as abnormal, resulting in a sensitivity of 92.9%. In the case of venous insufficiency, accurate evaluations were made for 392 cases, yielding a sensitivity of 97.5%. In the analysis using Grad-CAM, it was observed that the DenseNet121 program accurately recognized and analyzed regions indicating abnormal perfusion in photographs with compromised flap perfusion (Figure 4).

### Clinical Application

Based on FS-Net for segmentation and DenseNet 121 for grading, we developed an application named FLAPMATE (FLAP Monitoring Through AI Technology and Expertise). The application was developed using Flutter, version 3.13, to ensure cross-platform functionality, supporting both Android and iOS systems. The application maintained a compact size of 26.5 MB by using a server-client architecture, minimizing local storage requirements. Data processing to generate results after photo capture takes less than a second, with potential slight delays based on Wi-Fi conditions. We used this application for 10 patients. Following the existing monitoring protocol, we adjusted the monitoring interval to every 3 hours for the first 2 postoperative days and then every 6 hours thereafter. In total, 143 automatic monitoring sessions were conducted, with each patient undergoing between 12 and 19 sessions. The automatic monitoring proceeded smoothly, with most sessions being completed without triggering any alarms (Video). Alarms due to abnormalities occurred 3 times for 1 patient. When this patient was examined by the medical team, mild hyperemia of the flap was observed; however, no other signs suggesting flap perfusion compromise were found, and no specific management was conducted. After those events, flap color returned to normal and no further alarms were triggered. Eventually, all patients exhibited good flap conditions, and no perfusion-related complications occurred.

**Video.** Operational Process of the FLAPMATE Application for Automated Monitoring This video shows the operational process of the FLAP Monitoring Through AI Technology and Expertise (FLAPMATE) application during the monitoring procedure, demonstrating successful segmentation even in very small-sized flaps. The results from the grading model, evaluated based on images obtained through automatic segmentation of captured images, indicated a normal perfusion status, aligning with the clinical findings.

## Discussion

The present study has developed a novel free flap monitoring system aimed at alleviating the burden on medical staff for frequent flap monitoring while ensuring reliable results. Using an AI-based deep learning technique, we created a model capable of accurately segmenting flaps from images and developed a grading model to detect abnormalities in flap perfusion. Furthermore, we integrated these programs to create a system and evaluated its feasibility in clinical settings to determine whether free flap monitoring can be automated with minimal human intervention.

As previously mentioned, there have been attempts to use AI for flap monitoring in several studies,^8,9,10^ demonstrating impressive performance with an area under the curve greater than 0.99, slightly surpassing our model. However, despite their pioneering work and high accuracy, the models still required additional clinical evaluations, such as temperature measurements, in which human involvement may be challenging to replace or significantly reduce. The novelty of our model lies in its automation of all monitoring processes, including photo capture, segmentation, and perfusion grading, thereby minimizing the need for human intervention.

The development of the segmentation model is pivotal for ensuring the functionality of this system in clinical settings because the entire system is initiated by automatically identifying flaps from captured images and the performance of the subsequent flap perfusion grading model could be adversely affected when flap segmentation is not accurate. We identified that the CNN-based FS-Net model demonstrated excellent performance, showing a Dice similarity coefficient of 0.970, notable for including images captured not only close to the flap but also slightly farther away, incorporating scenes with other objects.

In the development of the classification model, we observed that the most recent DenseNet121 model demonstrated the highest performance, yielding sensitivity and specificity both exceeding 90%. The evaluation included images processed through the segmentation model (FS-Net) and covered both arterial insufficiency and venous insufficiency cases. Considering the aim of our program to function as a sentinel, minimizing false negatives would be paramount. Given that most false-negative images were associated with segmentation errors, especially in cases involving small flaps for finger or toe defect coverage, upgrading the segmentation model might hold promise for enhanced performance. The relatively lower sensitivity in cases with arterial insufficiency may be attributed to a smaller dataset used for training or subtle differences that are challenging to discern in images. Efforts are needed to improve sensitivity in arterial insufficiency for future enhancements.

### Limitations

This model does have several limitations. The performance of the AI model improves with more training data, particularly abnormal data, suggesting a need for a larger dataset of abnormal images to enhance accuracy. In particular, the precision of approximately 0.5 in the test set of the classification model can be considered one of the limitations. This precision is due to primarily false positives caused by mild bruising or hematoma. Although most mild hematomas resolve without issue, some may threaten overall flap perfusion, warranting attention. In addition, our primary goal was to serve as a sentinel rather than replace clinical monitoring entirely; therefore, the current performance may be acceptable for reducing the burden of clinical monitoring. Nonetheless, efforts to improve precision and reduce false positives are necessary. As the model’s training data primarily comprised patients of Asian ethnicity, its performance in situations involving darker skin tones remains uncertain. In addition, the model may show limited performance when applied to flap images with diverse lighting conditions and different smartphone models. To ensure commercial viability in broader fields, it will be crucial to evaluate the model with patients of diverse skin colors and under different lighting conditions as well as with the use of various smartphone models. Moreover, the applicability of the model is limited to visible skin paddles, which poses challenges for buried or muscle flaps.

## Conclusions

This study introduces a novel free flap monitoring system capable of automatically segmenting flap appearance and grading its perfusion based on photographs. The system shows potential for effective monitoring with minimal use of clinician time, offering promising reliability. Although it may not match the meticulousness of clinical evaluation by experienced staff, it excels as a vigilant sentinel, autonomously assessing flap perfusion and alerting medical personnel to abnormalities, potentially reducing health care workload. Further prospective studies are needed to fully assess its performance and versatility.

## Notes

Supplement 1. **eTable 1.** Data Augmentation Parameters for Training the Classification Model **eTable 2.** Baseline Characteristics and Operation Related Information of the Patients Included in the Study **eTable 3.** Performance of the Flap Segmentation Models for 5-Fold Cross Validation **eTable 4.** Performance of the Models for Flap Perfusion Classification **eTable 5.** Evaluation of Performances of DenseNet121 by the Flap Type and the Defect Site **eFigure 1.** Photographs Taken in a Non-Standardized Manner From the Retrospective Cohort (Left) and Those in a Standardized Manner From the Prospective Cohort (Right) **eFigure 2.** Structure of the FS-Net **eFigure 3.** Examples of the Process From Raw Image to Segmented Image

Supplement 2.
Data Sharing Statement
