# Supplementary material for: Development of an Automated Free Flap Monitoring System Based on Artificial Intelligence
Source: JAMA Netw Open. 2024 Jul 26;7(7):e2424299. doi: 10.1001/jamanetworkopen.2024.24299 (PMC11282448; doi:10.1001/jamanetworkopen.2024.24299)
Supplement: Supplement 1. — eTable 1. Data Augmentation Parameters for Training the Classification Model eTable 2. Baseline Characteristics and Operation Related Information of the Patients Included in the Study eTable 3. Performance of the Flap Segmentation Models for 5-Fold Cross Validation eTable 4. Performance of the Models for Flap Perfusion Classification eTable 5. Evaluation of Performances of DenseNet121 by the Flap Type and the Defect Site eFigure 1. Photographs Taken in a Non-Standardized Manner From the Retrospective Cohort (Left) and Those in a Standardized Manner From the Prospective Cohort (Right) eFigure 2. Structure of the FS-Net eFigure 3. Examples of the Process From Raw Image to Segmented Image [file jamanetwopen-e2424299-s001.pdf]

Supplementary Online Content

Kim JS, Lee SM, Kim DE, et al. Development of an automated free flap monitoring system based on artificial intelligence. *JAMA Netw Open.* 2024;7(7):e2424299. doi:10.1001/jamanetworkopen.2024.24299

eTable 1. Data Augmentation Parameters for Training the Classification Model

eTable 2. Baseline Characteristics and Operation Related Information of the Patients Included in the Study

eTable 3. Performance of the Flap Segmentation Models for 5-Fold Cross Validation

eTable 4. Performance of the Models for Flap Perfusion Classification

eTable 5. Evaluation of Performances of DenseNet121 by the Flap Type and the Defect Site

eFigure 1. Photographs Taken in a Non-Standardized Manner From the Retrospective Cohort (Left) and Those in a Standardized Manner From the Prospective Cohort (Right)

eFigure 2. Structure of the FS-Net

eFigure 3. Examples of the Process from Raw Image to Segmented Image

This supplementary material has been provided by the authors to give readers additional information about their work.

© 2024 Kim JS et al. *JAMA Network Open.*

eTable 1. Data Augmentation Parameters for Training the Classification Model

**Augmentation Type**	**Parameter**	**Explanation**
Flip	Horizontal	Flips 50% of the data randomly
	Vertical	Flips 50% of the data randomly
Brightness	Delta	Randomly adjusts brightness by selecting a delta (0 – 0.25), where higher values increase brightness
Contrast	Contrast factor	Randomly adjusts contrast by selecting a contrast factor (0.5 – 2.0), which modifies the intensity of the pixels, making the image either lighter or darker

© 2024 Kim JS et al. *JAMA Network Open.*

eTable 2. Baseline Characteristics and Operation Related Information of the Patients Included in the Study

	**Total** (n=305)	**Retrospective** (n = 203)	**Prospective** (n=102)
**Patient demographics**			
Age (median, range)	62 (8-86)	62 (8-86)	62 (13-95)
Sex			
Male	178 (58.4%)	124 (61.0%)	54 (52.9%)
Female	127 (41.6%)	79 (38.9%)	48 (47.1%)
Race			
Asian	302 (99.0%)	201 (99.0%)	101 (99.0%)
Caucasian	3 (1.0%)	2 (1%)	1 (1.0%)
Defect site			
Head and neck	62 (20.3%)	44 (21.7%)	18 (17.6%)
Trunk	66 (21.6%)	52 (25.6%)	14 (13.7%)
Extremity	177 (58.0%)	107 (52.7%)	70 (68.6%)
**Operation-related**			
Flap used			
ALT flap	144 (47.2%)	95 (40.8%)	49 (48.0%)
TDAP flap	48 (15.7%)	35 (15.0%)	13 (12.7%)
LD MC flap	45 (14.8%)	34 (14.6%)	11 (10.8%)
SCIP flap	42 (13.8%)	20 (8.6%)	22 (21.6%)
DIEP flap	25 (8.2%)	21 (9.0%)	4 (3.9%)
RASP flap	8 (2.6%)	8 (3.4%)	0

© 2024 Kim JS et al. *JAMA Network Open.*

Radial forearm free flap	7 (2.3%)	7 (3.0%)	0
Fibular free flap	3 (1.0%)	3 (1.3%)	0
DSAP flap	3 (1.0%)	3 (1.3%)	0
Others	10 (3.3%)	7 (3.0%)	3 (2.9%)

ALT, anterolateral thigh; TDAP, thoracodorsal artery perforator; LDMC, latissimus dorsi myocutaneous; SCIP, superficial circumflex iliac artery perforator, DIEP, deep inferior epigastric perforator; DSAP; dorsal scapular artery perforator

© 2024 Kim JS et al. *JAMA Network Open.*

eTable 3. Performance of the Flap Segmentation Models for 5-Fold Cross Validation

Models	Jaccard Index (mean ± SD)	Dice Coefficient (mean ± SD)	Specificity (mean ± SD)	Sensitivity (mean ± SD)
VGG16-U-Net	0.913±0.007	0.955±0.004	0.987±0.002	0.956±0.004
U-Net	0.921±0.004	0.959±0.002	0.988±0.002	0.959±0.004
FS-Net	0.942±0.019	0.970±0.010	0.992±0.004	0.970±0.010

SD; standard deviation

© 2024 Kim JS et al. *JAMA Network Open.*

eTable 4. Performance of the Models for Flap Perfusion Classification

Model	AUC (95% CI)	Sensitivity (95% CI)	Specificity (95% CI)	Specificity (at sensitivity 0.9)
VGG16	0.924 (0.908-0.939)	0.78 (0.744-0.815)	0.961 (0.955-0.966)	0.677
Custom CNN	0.934 (0.923-0.946)	0.9 (0.873-0.926)	0.852 (0.842-0.863)	0.852
InceptionV3	0.94 (0.929-0.951)	0.79 (0.753-0.826)	0.956 (0.950-0.961)	0.759
ResNet50	0.943 (0.930-0.955)	0.908 (0.884-0.933)	0.857 (0.847-0.867)	0.863
DenseNet121	0.96 (0.950-0.969)	0.91 (0.886-0.934)	0.910 (0.903-0.918)	0.909

© 2024 Kim JS et al. *JAMA Network Open.*

eTable 5. Evaluation of Performances of DenseNet121 by the Flap Type and the Defect Site

Subgroup	Rate of abnormal cases (%)	AUC (95% CI)	Sensitivity (95% CI)	Specificity (95% CI)	Sensitivity*	Specificity*
Flap type						
ALT	6.2%	0.937(0.911-0.961)	0.839(0.771-0.906)	0.919(0.907-0.931)	0.737	0.974
LD MC & TDAP	1.3%	0.924(0.826-0.980)	0.909(0.700-1.000)	0.899(0.878-0.920)	0.818	0.936
SCIP	12.0%	0.963(0.952-0.973)	0.982(0.967-0.996)	0.794(0.766-0.822)	0.933	0.794
Others	5.2%	0.959(0.940-0.977)	1.000(1.000-1.000)	0.861(0.831-0.892)	0.897	0.904
Defect site						
Head & neck	8.4%	0.943(0.923-0.963)	0.849(0.794-0.903)	0.929(0.912-0.945)	0.831	0.939
Trunk	2.5%	0.997(0.993-1.000)	1.000(1.000-1.000)	0.966(0.952-0.979)	0.941	0.987
Extremity	9.0%	0.967(0.959-0.974)	0.980(0.963-0.996)	0.874(0.860-0.887)	0.953	0.892

* The results were calculated based on the optimal threshold presented in Supplementary Table 4.

© 2024 Kim JS et al. *JAMA Network Open.*

eFigure 1. Photographs Taken in a Non-Standardized Manner From the Retrospective Cohort (Left) and Those in a Standardized Manner From the Prospective Cohort (Right)

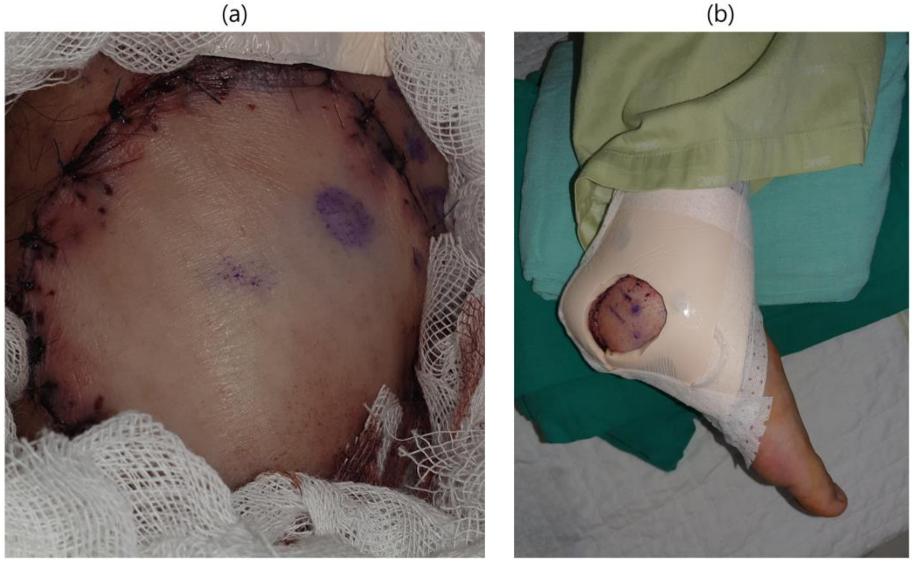

© 2024 Kim JS et al. *JAMA Network Open.*

eFigure 2. Structure of the FS-Net

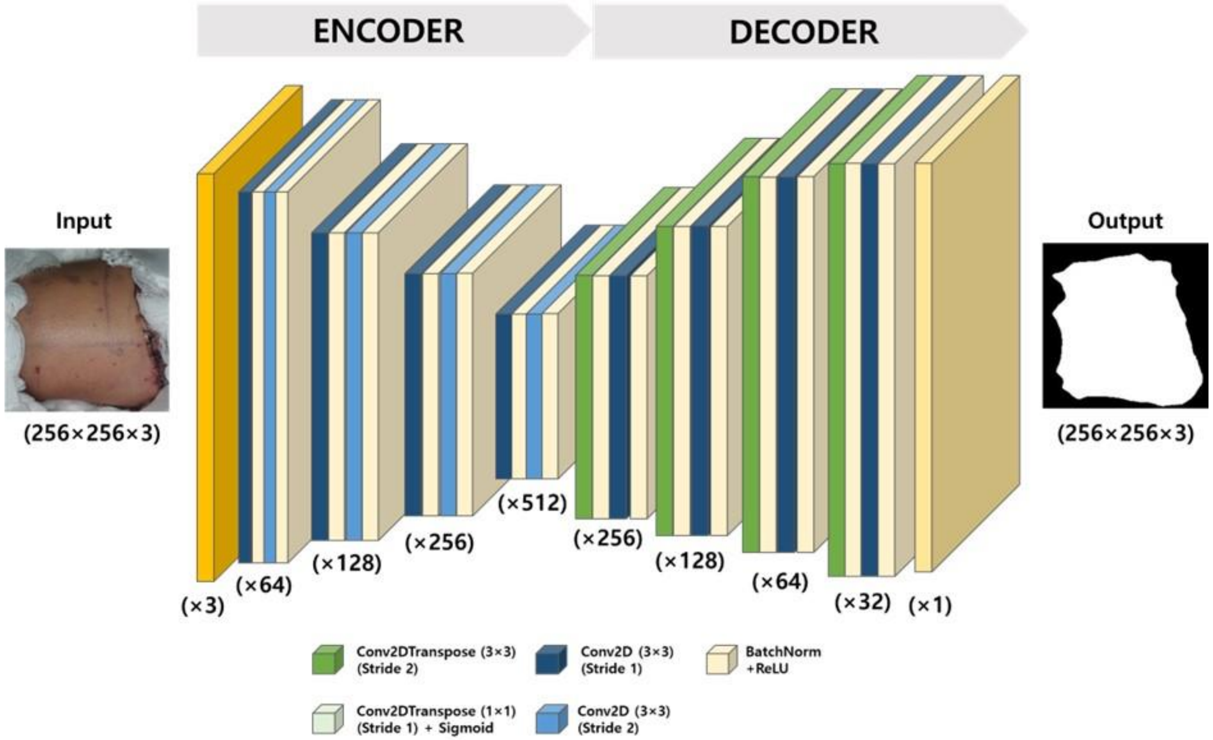

© 2024 Kim JS et al. *JAMA Network Open.*

eFigure 3. Examples of the Process from Raw Image to Segmented Image. (A) Origin flap images, (B) Manual annotation by expert (Ground truth), (C) Mask predicted by FS-Net, (D) Segmentation results by FS-Net

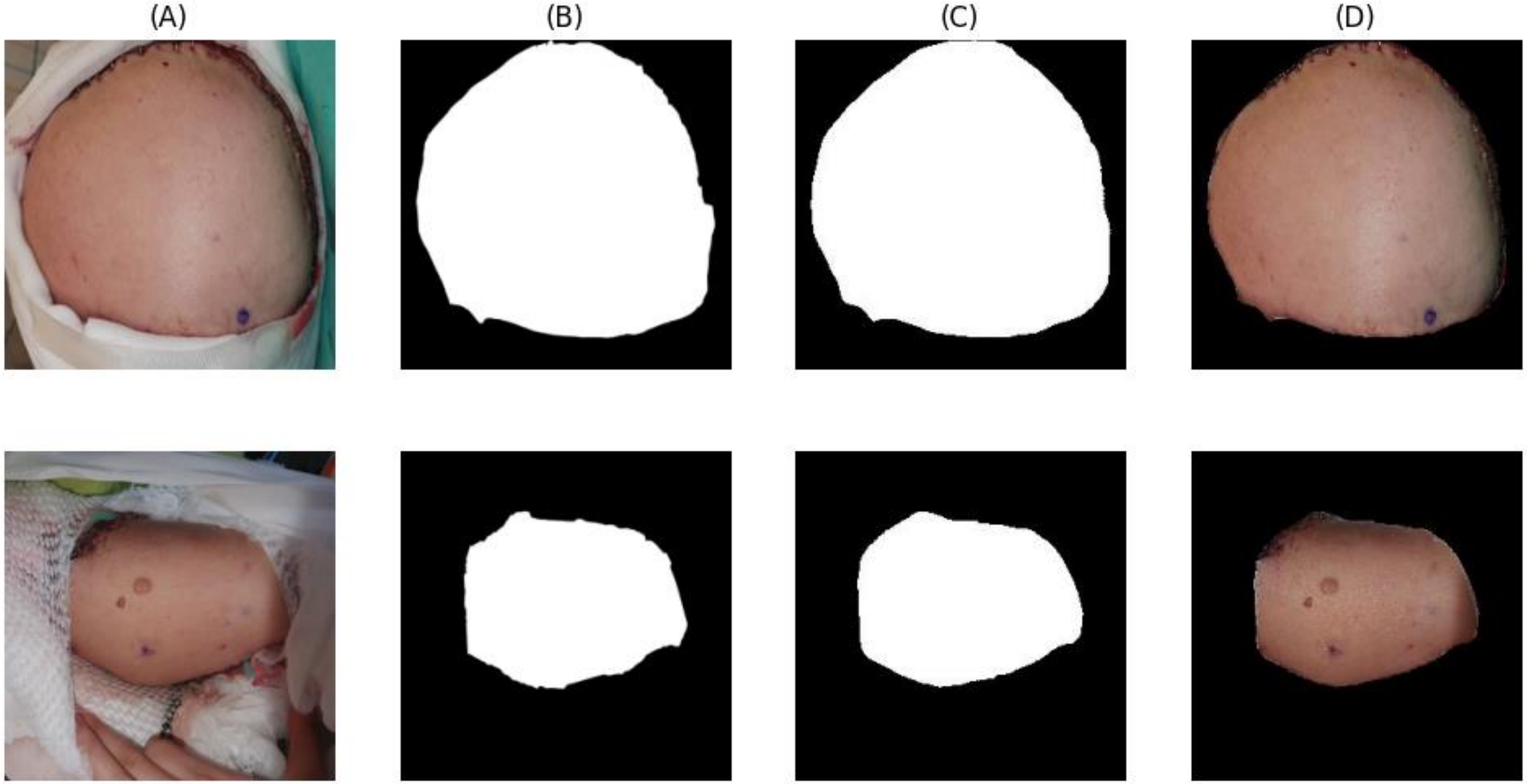

© 2024 Kim JS et al. *JAMA Network Open.*
