# Supplementary material for: Development of an Automated Free Flap Monitoring System Based on Artificial Intelligence
Source: JAMA Netw Open. 2024 Jul 26;7(7):e2424299. doi: 10.1001/jamanetworkopen.2024.24299 (PMC11282448; doi:10.1001/jamanetworkopen.2024.24299)
Supplement: Supplement 2. — Data Sharing Statement [file jamanetwopen-e2424299-s002.pdf]

Data Sharing Statement

Kim. Development of an Automated Free Flap Monitoring System Based on Artificial Intelligence. *JAMA Netw Open*. Published July 26, 2024.
doi:10.1001/jamanetworkopen.2024.24299

Data
Data available: No
